# Case report: Pulmonary sarcomatoid carcinoma demonstrating rapid growth on follow-up CT

**DOI:** 10.3389/fonc.2024.1393203

**Published:** 2024-07-08

**Authors:** Li Tu, Hong Xie, Lianshan Zhan, Yushi Yang, Tingting Chen, Na Hu, Xiaojun Du, Shi Zhou

**Affiliations:** ^1^ General Practice Department, The Affiliated Hospital of Guizhou Medical University, Guiyang, Guizhou, China; ^2^ Radiology Department, The Affiliated Hospital of Guizhou Medical University, Guiyang, Guizhou, China; ^3^ Nuclear Medicine Department, Guiqian International General Hospital, Guiyang, Guizhou, China; ^4^ Pathology Department, The Affiliated Hospital of Guizhou Medical University, Guiyang, Guizhou, China; ^5^ Thoracic Surgery Department, The Affiliated Hospital of Guizhou Medical University, Guiyang, Guizhou, China; ^6^ Interventional Radiology Department, The Affiliated Hospital of Guizhou Medical University, Guiyang, Guizhou, China

**Keywords:** lung cancer, pulmonary sarcomatoid carcinoma, tumor growth rate (TGR), tumor volume doubling time, case report

## Abstract

**Background:**

The tumor growth rate and tumor volume doubling time are crucial parameters in diagnosing and managing lung lesions. Pulmonary sarcomatoid carcinoma (PSC) is a unique and highly malignant subtype of lung cancer, with limited documentation on its growth feature. This article aims to address the gap in knowledge regarding a PSC’s growth patterns by describing the characteristics of a confirmed case using computed tomography, thereby enhancing the understanding of this rare disease.

**Case presentation:**

A 79-year-old man was transferred to our center presenting with a mild cough, blood-tinged sputum, and a malignant nodule in the left upper lobe. Chest CT revealed a solid nodule in the left upper lobe. A follow-up CT ten days later showed a significant increase in the size of the nodule, accompanied by ground-glass opacity in the surrounding lung. The rapid preoperative growth of the nodule suggested a non-neoplastic lesion, and intraoperative frozen pathology also considered the possibility of tuberculosis. Subsequently, a left upper apical-posterior segment (S1 + 2) resection was performed. Postoperative tumor pathology confirmed the diagnosis of pulmonary sarcomatoid carcinoma with extensive giant cell carcinoma and necrosis. Immunohistochemistry indicated approximately 60% PD-L1 positive and genetic testing revealed a MET mutation. The patient was discharged with oral crizotinib targeted therapy, and his condition remained stable postoperatively. The patient is currently undergoing regular follow-up at our hospital, with no evidence of distant metastasis or recurrence.

**Conclusion:**

Pulmonary sarcomatoid carcinoma can exhibit rapid tumor growth on imaging, and PSC should be considered in the differential diagnosis for lesions that present with a fast growth rate. Timely and appropriate treatment for PSC may lead to a good prognosis.

## Introduction

Lung cancer remains the most prevalent malignant tumor globally, with a spectrum of pathological subtypes that exhibit distinct biological behaviors, treatment responses, and outcomes (1). Among these, pulmonary sarcomatoid carcinoma (PSC) stands out as a rare and aggressive variant of non-small cell lung cancer (NSCLC), constituting only 0.1%-0.4% of lung malignancies (2). The World Health Organization’s classification of tumors classified PSC into five pathological types (3, 4), and each type has its own characteristics and clinical behaviors. Given the poor prognosis associated with most PSCs and the potential for tailored therapies to enhance survival rates (5, 6), there is an urgent need to deepen our understanding of this rare disease.

The tumor growth rate is a critical determinant in lung cancer management, reflecting the tumor’s aggressiveness and guiding clinical decisions on follow-up intervals and treatment strategies. Rapid growth rates and short doubling times are indicative of high malignancy, often necessitating immediate intervention (7), while slower rates may allow for a more deliberate approach to treatment selection (8). Pulmonary tumors and tumor-like lesions exhibit a spectrum of growth rates, with pulmonary nodules being a prevalent presentation in the early stages of lung cancer. This variability in growth kinetics is a key factor informing the differential follow-up intervals for various types of pulmonary nodules in clinical practice. Consequently, it forms a substantial basis for the development of current clinical guidelines, consensus, and expert opinions regarding the management of pulmonary nodules (9, 10).

While there is a wealth of literature on tumor growth rate of lung cancer, the specific growth dynamics of PSC are understudied (11, 12). Little is known about growth pattern of PSC, and there is a lack of reports on the tumor volume doubling time and tumor growth rate of PSC (13, 14). Our report addresses this gap by presenting a detailed analysis of the tumor growth rate and volume doubling time of a PSC case from serial follow-up CT scans. This case highlights the potential for rapid growth in PSC, which is uncommon in lung cancer. By elucidating the growth kinetics of this rare tumor, we aim to enrich the sparse literature on PSC and contribute to a more nuanced understanding of lung lesions that exhibit rapid tumor growth.

## Case presentation

**Figure 1:**
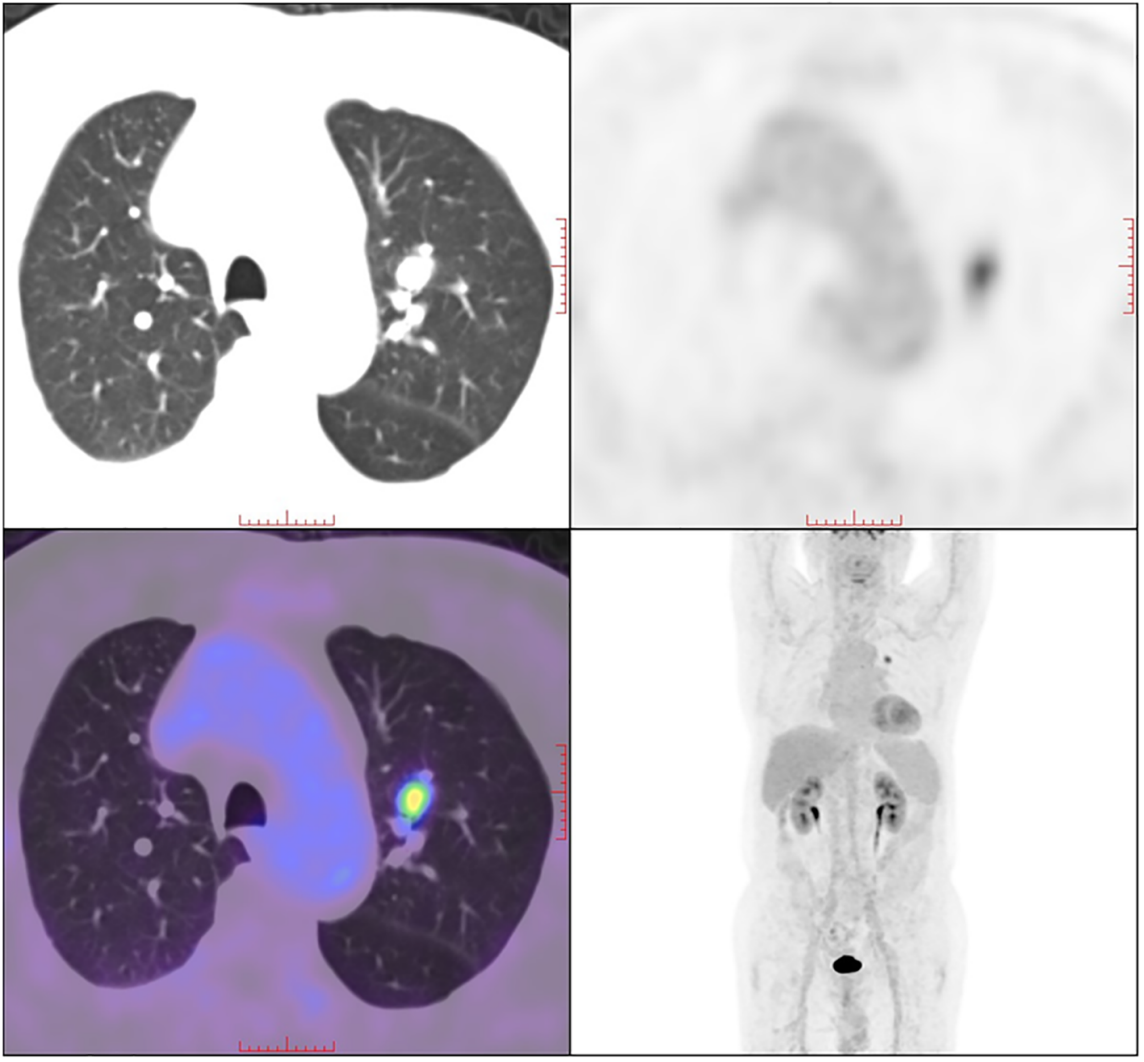
18F-FDG PET/CT scan of the patient. A solid nodule in the left upper lobe with increased 18F-FDG uptake was detected.

A 79-year-old male presented with left chest pain for over 10 days, accompanied by a cough and phlegm for over 7 months. The patient developed a mild cough and phlegm without a clear cause, and recently blood-tinged sputum, which was dark red and occasionally bright red, averaging about 10ml per day, was noticed. Imaging from another hospital suggested a nodule in the left upper lobe. The patient’s PET-CT in August, 2021 showed increased metabolism of a nodule in the apical posterior segment of the left upper lobe, the nodule measures approximately 12×10 mm in size, it has an irregular shape, with clear margins. The standardized uptake value of the nodule is approximately 6.7 (**Figure 1**), indicative of a malignancy.

Upon admission, the patient was in good general condition with temperature at 36°C, heart rate at 80 beats/min, and respiratory rate at 20 breaths/min, he walked into the hospital, and his blood pressure was 136/88mmHg after taking medication for hypertension. The patient has an over 40 years history of smoking, about one pack per day, and has quit for over 15 years; a 50-year history of alcohol consumption, with 2 Liang (approximately 100ml) of white liquor daily, and has quit for 7 months. Pulmonary Function earlier suggested mild mixed restrictive and obstructive pulmonary ventilation disorder and small airway obstruction.

**Figure 2:**
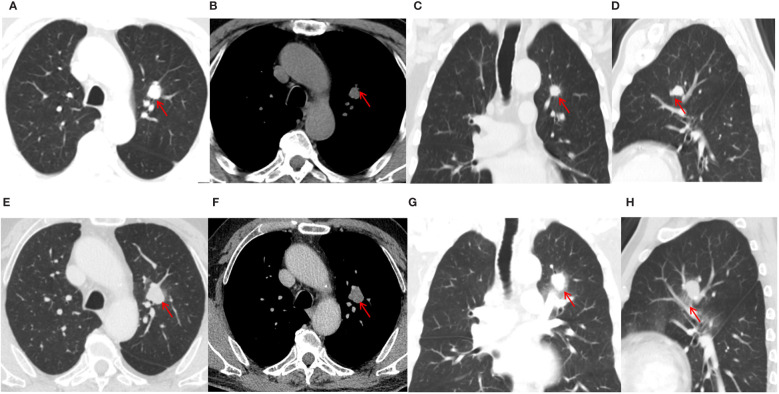
Chest CT of the patient. Plain CT on November 18, 2021 **(A–D)** and contrast enhanced CT on November 28 **(E–H)**. Patches of ground glass opacity can be observed around the lesion on image **(G, H)**.

Chest CT of the patient on November 18, 2021 showed a left upper lung nodule measuring approximately 18×13mm (**Figures 2A–D**), the nodule has an irregular shape with a lobulated appearance. The margins are characterized by speculated protrusions and multiple long spicules. Vascular structures appear to traverse within the nodule. A contrast-enhanced CT 10 days later detected an obvious increase in the size of the nodule, measuring about 23×20mm, with clear boundaries and moderate uniform enhancement, and multiple patchy ground-glass opacity around the lesion (**Figures 2E–H**). And the volume of the nodule increased from 546 cubic millimeters to 2024 cubic millimeters within 10 days.

**Figure 3:**
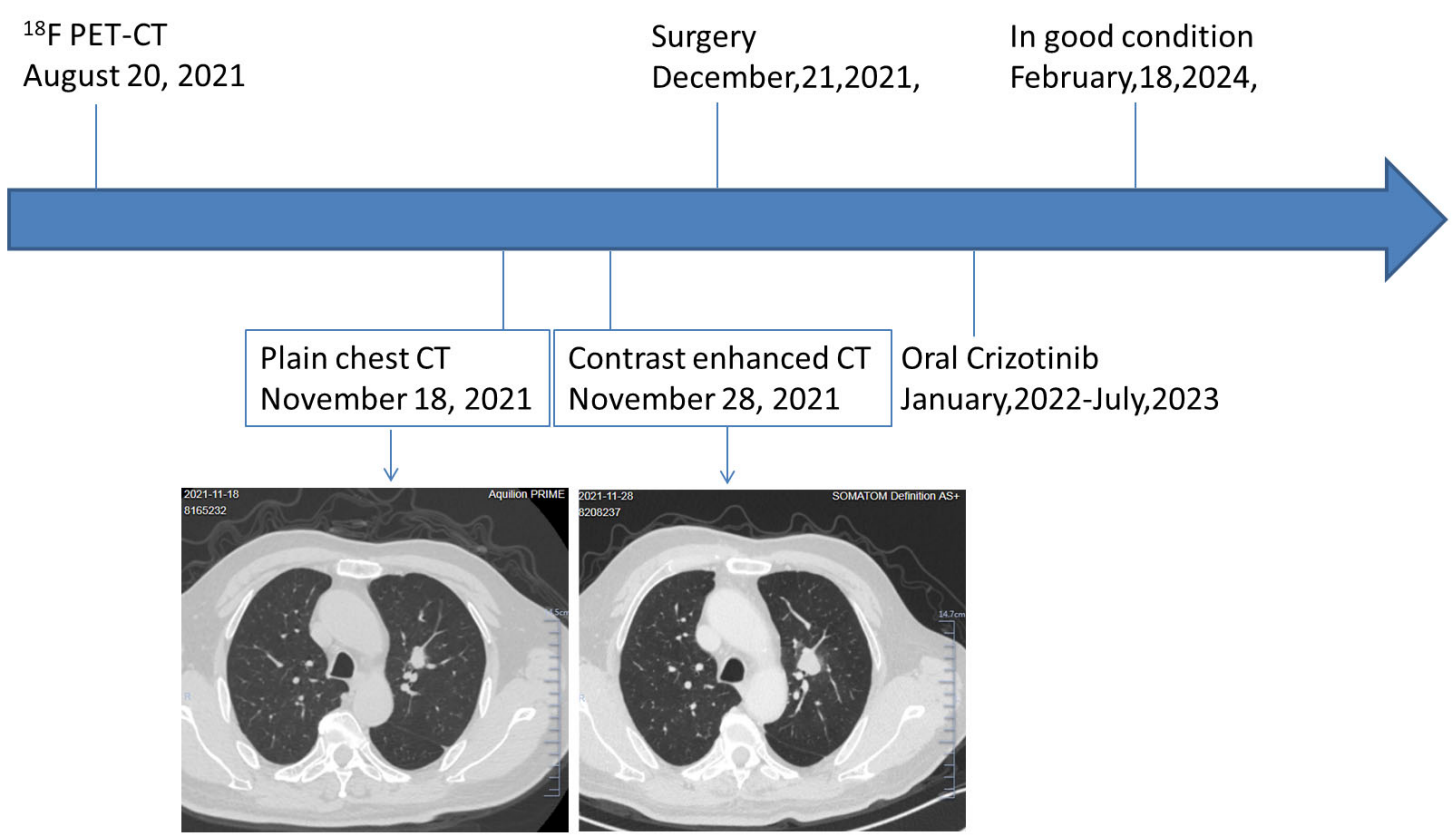
Timeline of the patient’s diagnosis and treatment.

The rapid volume increase in such a short period with surrounding ground-glass opacity of the left upper lung nodule did not match the growth feature of common malignant tumors in the lungs, and preoperative imaging by a chest radiologist with over 30 years of diagnosing experience considered non-neoplastic lesions. The patient underwent a thoracoscopic resection of the lesion within the left upper lobe’s inherent segment, employing a complex pulmonary segmentectomy of the apical-posterior segment (S1 + 2). The resected lung segment volume was 11x7.5x2cm, the bronchial diameter was 0.8cm, and a nodule was found 1cm under the pleura, 2cm from the bronchial cut end. The cut surface was gray-brown and crumbly, with a nodule diameter of 2cm. No involvement of the bronchial cut end was seen, and a lymph node was found next to the bronchus. Intraoperative pathology showed a large area of necrosis and hemorrhage in the resected lung tissue, surrounded by a small number of atypical cells, and tuberculosis was considered during the operation **Figure 3**

**Figure 4:**
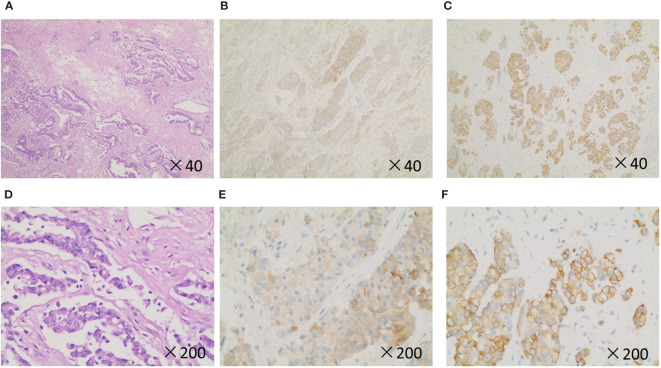
Histopathology and immunohistochemistry of the patient. **(A, D)**, H&E stain. **(B, E)**, Syn. **(C, F)**, CK-19. Original magnification: ×40 and × 200.

Postoperative immunohistochemistry after routine pathological sampling suggested atypical large cells and giant cells proliferating around the extensive lesion, distributed in small patches, clusters, or glandular distribution. Immunohistochemistry results of atypical cells were CK (+), EMA (+), Vim (+), CK7 (+), CK5/6 (minority+), P63 (minority +), P40 (minority +), NapsinA (minority +), TTF-1 (minority +), CD56 (-), CgA (-), Syn (-), CD68 (-), LCA (-). PDL1 (about 60% positive), Ki-67 (about 60% positive) (**Figure 4**). The final pathology diagnosis was sarcomatoid carcinoma with extensive necrosis (giant cell carcinoma with necrosis), and the tumor’s pathological stage is classified as T1N0M0, genetic testing results revealed a MET gene mutation in the patient. After discharge, the patient was given an oral Crizotinib capsule for targeted therapy. The patient underwent follow-up at 3-month interval in the first 18 months during the treatment, and he experienced any serious complications. Currently, the patient regularly visits our outpatient clinic for follow-up every 3 to 6 months, and no signs of recurrence or metastasis have been detected. At the last follow-up, he was in good general condition, and he took daily exercises for half an hour.

## Discussion

This case reports an elderly male pulmonary sarcomatoid carcinoma patient with a unique radiological growth feature. The imaging data were complete, and the nodule showed rapid growth on follow-up CT. Both preoperative and intraoperative frozen pathology misdiagnosed the lesion as non-neoplastic diseases, which was ultimately confirmed as PSC through surgical pathology. As a rare pathological type of NSCLC, PSC is known for its strong invasiveness and poor prognosis (13, 15). PSCs are poorly differentiated, contain sarcomatous or sarcoma-like components, grow rapidly, and have a high degree of vascular invasion (15, 16). Histologically, PSC is a tumor with atypia, transforming from the typical epithelial appearance of cancer to spindle-shaped cells or bizarre tumor giant cells resembling sarcoma (3). This disease occurs in 60%-80% of cases in males, with a peak incidence age of 70 years, and smoking is the main risk factor (6). This patient, aged 79, had a history of smoking for decades and had quit for over 10 years.

Tumor growth feature is one of the core characteristics of many tumors, it is closely related to the aggressiveness, pathological type, and prognosis of the tumor (9), directly affecting the management and treatment of patients (11). In this case, the tumor volume doubling time is about 6 days, and this means the lesion doubles in volume every 6 days (17). This growth rate is unexpectedly rapid, surpassing the growth rates of common documented lung cancer types such as adenocarcinoma, squamous cell carcinoma, and small cell carcinoma found in many pieces of literature (8, 18). In most types of lung cancer, the tumor volume doubling time ranges from tens to hundreds of days (19, 20). As far as we are concerned, only one case of pleomorphic carcinoma has been reported presenting with rapid growth in English literatures (21), but the detailed tumor growth rate and tumor volume doubling time were not provided in their report.

The rapid increase in volume in this case provided direct evidence of the aggressiveness of PSC (7). In the provided case, the follow-up period before treatment was marked by a striking increase in the size of the lung nodule, with a volume expansion from 546 mm³ to 2024 mm³ within a mere 10 days. The swift volumetric change underscores the unpredictable and aggressive behavior of pulmonary sarcomatoid carcinoma, and this phenomenon might be a sign of malignant changes, highlighting the importance of close monitoring or timely intervention. As this is only a phenomenon from a case report, more investigation is needed in the future.

Lesions with similar rapid growth rates in the lung are more commonly seen in inflammatory lesions (22, 23), and it is reported that rapid growth of pulmonary nodules on CT scans is not a predictor of malignancy. This might be one reason for the misdiagnosis of CT preoperatively in this case. PSC should be included in the differential diagnosis of lesions with rapid growth in the lung. The patient’s follow-up CT showed ground-glass opacity around the lesion, and it was confirmed to be necrosis combined with hemorrhage postoperatively. Ninety percent of PSC cases exhibit vascular invasion, which may be related to the ease of recurrence and metastasis, and this might be related to the patient’s symptoms of hemorrhage in the area surrounding the nodule on pathology.

There is no established best treatment and follow-up strategy for PSC, and the most widely used treatment is a comprehensive treatment strategy primarily based on surgery (13, 24). Surgery can improve the survival of PSC patients, especially for those with early-stage tumors (25). This case supports that timely surgery might lead to a good prognosis in PSC. Although there was not much evidence, the patient was given targeted therapy according to the gene result (14, 16, 26). Over-expression of PD-L1 is observed in more than half of pulmonary sarcomas, and the PD-1 inhibitor is gradually becoming a promising new method for the treatment of PSC patients (26, 27). A multidisciplinary comprehensive approach, integrating clinical staging, pathology, genetic testing, and other disciplines, may help in selecting more appropriate and precise treatments, and might be an important direction for improving patient prognosis (26, 28).

In conclusion, this case indicates that PSC can exhibit rapid growth on imaging in the early stage, accompanied by peripheral bleeding. When middle-aged or elderly men with a long history of smoking are found to have upper lobe pulmonary nodules with rapid growth, PSC should be considered in the differential diagnosis. Timely and appropriate treatment might lead to good prognosis for this rare but aggressive malignancy.

## Data availability statement

The raw data supporting the conclusions of this article will be made available by the authors, without undue reservation.

## Ethics statement

Ethical approval was not required for the study involving humans in accordance with the local legislation and institutional requirements. Written informed consent to participate in this study was not required from the participants or the participants’ legal guardians/next of kin in accordance with the national legislation and the institutional requirements. Written informed consent was obtained from the individual(s) for the publication of any potentially identifiable images or data included in this article.

## Author contributions

LT: Conceptualization, Data curation, Funding acquisition, Writing – original draft. HX: Conceptualization, Data curation, Funding acquisition, Writing – original draft, Writing – review & editing, Supervision. LZ: Conceptualization, Data curation, Writing – original draft. YY: Conceptualization, Data curation, Writing – original draft. TC: Data curation, Writing – original draft. NH: Writing – review & editing. XD: Conceptualization, Data curation, Writing – review & editing. SZ: Conceptualization, Data curation, Writing – review & editing.
